# Soil microbial processes shaping seed performance: linking soil microbiomes to sustainable agriculture

**DOI:** 10.3389/fmicb.2026.1797362

**Published:** 2026-03-26

**Authors:** Einstein Mariya David, Theivasigamani Parthasarathi

**Affiliations:** 1 School of BioSciences and Technology (SBST), Vellore Institute of Technology, Vellore, Tamil Nadu, India; 2 VIT School of Agricultural Innovations and Advanced Learning (VAIAL), Vellore Institute of Technology, Vellore, Tamil Nadu, India

**Keywords:** nature-based solutions, plant microbe interactions, salinity stress, seed microbiome, soil microorganisms, soil seed microbiome continuum, soil sustainability, sustainable agriculture

## Abstract

Soil microorganisms are fundamental to soil sustainability, governing organic matter turnover, nutrient cycling, soil structure formation, and plant health regulation. In the context of accelerating soil degradation, climate change, and expanding agricultural salinization, understanding how soil microbial communities contribute to ecosystem resilience is crucial for sustainable soil management. Although rhizosphere and plant nutrition roles are well recognized, their influence across plant life cycles and generations remains insufficiently integrated. This Review synthesizes recent advances to propose the soil seed microbiome continuum as a unifying concept linking soil microbial processes to seed quality, early plant establishment, and crop stress tolerance under salinity stress. Unlike existing microbiome salinity reviews that predominantly focus on rhizosphere interactions or microbial inoculants under salt stress, this review advances an integrative soil seed continuum framework that connects soil ecological processes, microbial transmission, and seed associated microbiomes with a transgenerational context. We discuss how this ecosystem acts as a dynamic reservoir of beneficial and stress-adapted microorganisms that are selectively recruited by plants, transmitted through plant associated pathways, and ultimately incorporated into developing seeds. Under saline conditions, ecological filtering favors halotolerant microbial taxa that stabilize soil functions, and enhancing plant stress tolerance, with potential transgenerational benefits mediated through seed-associated microbiomes. The evidence from soil microbial ecology, plant microbe interactions, and emerging microbiome-enabled technologies, this review highlights the role of soil microorganisms as biological connectors between soil sustainability and crop performance. We further discuss implications for reduced chemical inputs, yield stability, nature-based restoration, and contributions to the United Nations Sustainable Development Goals. Positioning soil microorganisms within a soil seed continuum offers new perspectives for managing soil biodiversity and functionality, reinforcing their central role in sustainable agriculture and resilient soil ecosystems. This integrative perspective provides a strategic foundation for developing microbiome informed soil management approaches aimed at enhancing long term crop performance under increasing salinization and climate change.

## 1 Introduction

Soil microorganisms constitute the biological foundation of soil sustainability and are increasingly recognized as central regulators of agroecosystem resilience, productivity, and environmental stability. Diverse microbial assemblages drive essential processes including organic matter decomposition, nutrient cycling, soil aggregation, and pathogen suppression. Together, these functions underpin soil fertility and structural stability, enabling soils to sustain plant growth and buffer environmental disturbances (Chen et al., 2024; Streletskii et al., 2024). However, intensive agricultural practices, excessive reliance on synthetic fertilizers, land degradation, and accelerating climate change are progressively eroding soil microbial diversity and functional capacity, posing serious threats to long-term soil health and global food security (Futa et al., 2024).

Among the major abiotic constraints affecting agricultural soils, salinity represents one of the most pervasive and rapidly expanding challenges worldwide. Soil salinization alters soil physicochemical properties, disrupts microbial activity, limits nutrient availability, and imposes osmotic and ionic stress on plants, ultimately resulting in substantial yield losses across diverse cropping systems (Shrivastava and Kumar, 2015; Jabbar et al., 2025) Conventional mitigation approaches, including the development of salt-tolerant cultivars and the application of chemical soil amendments, are often resource-intensive, and slow to deliver durable outcomes. These limitations highlight the urgent need for low-input, nature-based strategies that simultaneously restore soil functionality and enhance crop tolerance to salinity stress.

Soil microorganisms offer such a biologically grounded solution by acting as critical mediators between soil processes and plant stress responses. Increasing evidence demonstrates that plant growth promoting rhizobacteria, arbuscular mycorrhizal fungi, actinobacteria, and other beneficial microbial groups improve plant performance under saline conditions through multiple mechanisms, including enhanced nutrient acquisition, regulation of ion homeostasis, production of phytohormones and osmoprotectants, activation of antioxidant defenses, and improvement of soil structural properties (Bargaz et al., 2018; Marzouk et al., 2025; SLITI et al., 2025). Notably, salinity does not uniformly suppress soil microbial communities; rather, it acts as an ecological filter that selectively enriches halotolerant and stress-adapted taxa capable of maintaining soil processes and supporting plant growth under adverse conditions (Tang et al., 2023). Such enrichment frequently includes halotolerant bacterial groups such as *Bacillus, Halomonas*, *Pseudomonas*, *Actinobacteria*, and salt adapted fungal taxa including *Trichoderma* and *Mortierella.*

Recent advances in soil microbial ecology further reveal that the influence of soil microorganisms extends beyond the rhizosphere and root compartments into plant reproductive tissues. Seeds, once considered microbiologically inert, are now recognized as repositories of distinct and functionally relevant microbial communities that influence germination, seedling vigor, stress tolerance, and early plant establishment (Romão et al., 2025). Accumulating evidence indicates that many seed-associated microorganisms originate from soil, entering plants via the rhizosphere and endosphere and being selectively transmitted to developing seeds through vascular or floral pathways. This sequential microbial transfer supports the concept of a soil seed continuum, in which soil microbial communities represent the primary ecological and evolutionary source of seed microbiota.

The soil seed continuum is particularly relevant in the context of salinity stress and climate-resilient agriculture. Soil functions as a reservoir of stress-adapted microorganisms that can be recruited by plants and, in some cases, transmitted across generations through seeds, potentially conferring early-life advantages to seedlings exposed to saline environments. Because salinity stress is particularly severe during germination and early seedling establishment, seed associated microbiomes may provide osmotic buffering, ion homeostasis support, and activation of stress-responsive pathways before rhizosphere recruitment is fully established. Such microbially mediated, transgenerational stress tolerance represents a promising yet underexplored mechanism linking soil health, plant adaptation, and agricultural sustainability. While previous studies have independently examined soil microbiome functions, rhizosphere dynamics, or seed-associated microbial communities, an integrated synthesis positioning soil microorganisms as integrative drivers linking soil sustainability, seed microbiome assembly, and salinity tolerance remains conceptually underdeveloped.

Addressing this conceptual gap is increasingly urgent in light of escalating soil salinization, climate variability, and input-intensive agricultural systems. This review synthesizes recent advances in soil and seed microbiome research to conceptualize the soil seed continuum as a unifying framework for understanding microbe-mediated salinity tolerance. By integrating insights from soil microbial ecology, plant microbe interactions, and emerging microbiome-enabled agricultural approaches, this review highlights the potential of harnessing soil microorganisms to enhance soil sustainability, strengthen crop resilience, and support the transition toward sustainable agricultural systems.

## 2 Soil microorganisms as foundations of soil sustainability

Soil microorganisms constitute the biological foundation of soil health and sustainability, governing a wide array of biogeochemical and ecological processes that underpin agricultural productivity. Diverse microbial taxa including bacteria, fungi, archaea, and actinobacteria drive the decomposition of organic residues and regulate the transformation and mobilization of essential nutrients such as nitrogen, phosphorus, sulfur, and carbon. Through process such as nitrogen fixation, phosphate solubilization, mineral weathering, and organic matter turnover, soil microbiomes directly determine nutrient availability and soil fertility across agroecosystems (Jacoby et al., 2017; Chen et al., 2024; Wang et al., 2024).

Beyond nutrient cycling, soil microorganisms exert a profound influence on the physical architecture of soils. Microbial-derived extracellular polymeric substances, fungal hyphal networks, and biofilm formation promote soil aggregation, pore connectivity, and structural stability. These microbially mediated processes enhance water infiltration, retention, and aeration properties that are particularly critical under salinity stress, where osmotic imbalance and ion toxicity compromise soil function and plant growth (Islam et al., 2024; Iqbal et al., 2025). Stable soil structure further supports carbon sequestration and long-term soil resilience, reinforcing the role of microorganisms as invisible architects of sustainable soils. These functions are mediated by diverse bacterial and fungal groups whose activity and interactions determine soil structural integrity and nutrient dynamics.

Soil microorganisms also interact intimately with plants through complex and dynamic plant–microbe associations. Rhizobacteria and mycorrhizal fungi enhance plant nutrient acquisition by extending the effective root surface area, mobilizing poorly available nutrients, and modulating rhizosphere chemistry. Many beneficial microbes synthesize phytohormones, siderophores, and antimicrobial compounds that regulate plant growth, suppress pathogens, and improve stress tolerance. These interactions are highly sensitive to land-use practices, soil physicochemical properties, and climatic pressures, highlighting the responsiveness of soil microbiomes to both natural and anthropogenic drivers (Neale et al., 2024; Pandey and Saharan, 2025).

Under saline conditions, soil microbial communities are subjected to strong selective pressures that reshape their composition and functional potential. Salinity favors the enrichment of halotolerant and stress-adapted microbial taxa capable of maintaining metabolic activity under high osmotic stress and ionic imbalance. Such microorganisms contribute to plant salinity tolerance by improving nutrient uptake efficiency, producing osmoprotectants, regulating ion homeostasis, and mitigating oxidative stress. Recent metagenomic studies further demonstrate that salinity, especially when combined with other global change factors, drives functional diversification within soil microbiomes, underscoring their adaptive capacity under multiple environmental constraints (del Rodríguez Río et al., 2025).

Despite extensive characterization of microbial functions, important questions remain regarding the stability, context dependency, and scalability of these processes under field conditions. Increasingly, research highlights that soil functionality emerges from community-level interactions, ecological filtering, and functional redundancy rather than the presence of individual taxa alone. Understanding these dynamics is critical for translating microbial potential into predictable soil sustainability outcomes.

Importantly, stress-adapted soil microbiomes function as reservoirs of beneficial microorganisms that can be recruited by plants and transmitted across plant compartments. Recruitment is mediated by root exudate driven selection in the rhizosphere, followed by endophytic colonization and systemic movement through vascular tissues. Transmission to reproductive structures may occur via floral pathways or internal vascular transport, where additional ecological filtering shapes seed-associated communities. This soil-to-seed linkage positions soil microorganisms as foundational agents within the soil seed continuum.

## 3 The soil seed microbiome continuum: pathways of microbial transmission

Plants exist as holobionts whose performance and resilience are shaped not only by their genotype but also by the microbial consortia associated with different plant compartments. Increasing evidence supports the concept of a soil seed microbiome continuum, in which soil acts as the primary microbial reservoir, plants serve as ecological filters, and seeds function as vectors for microbial transmission across generations. Rather than being isolated niches, soil, rhizosphere, endosphere, and seeds are interconnected habitats linked through structured transmission pathways that determine microbial persistence, inheritance, and function.

**Figure 1:**
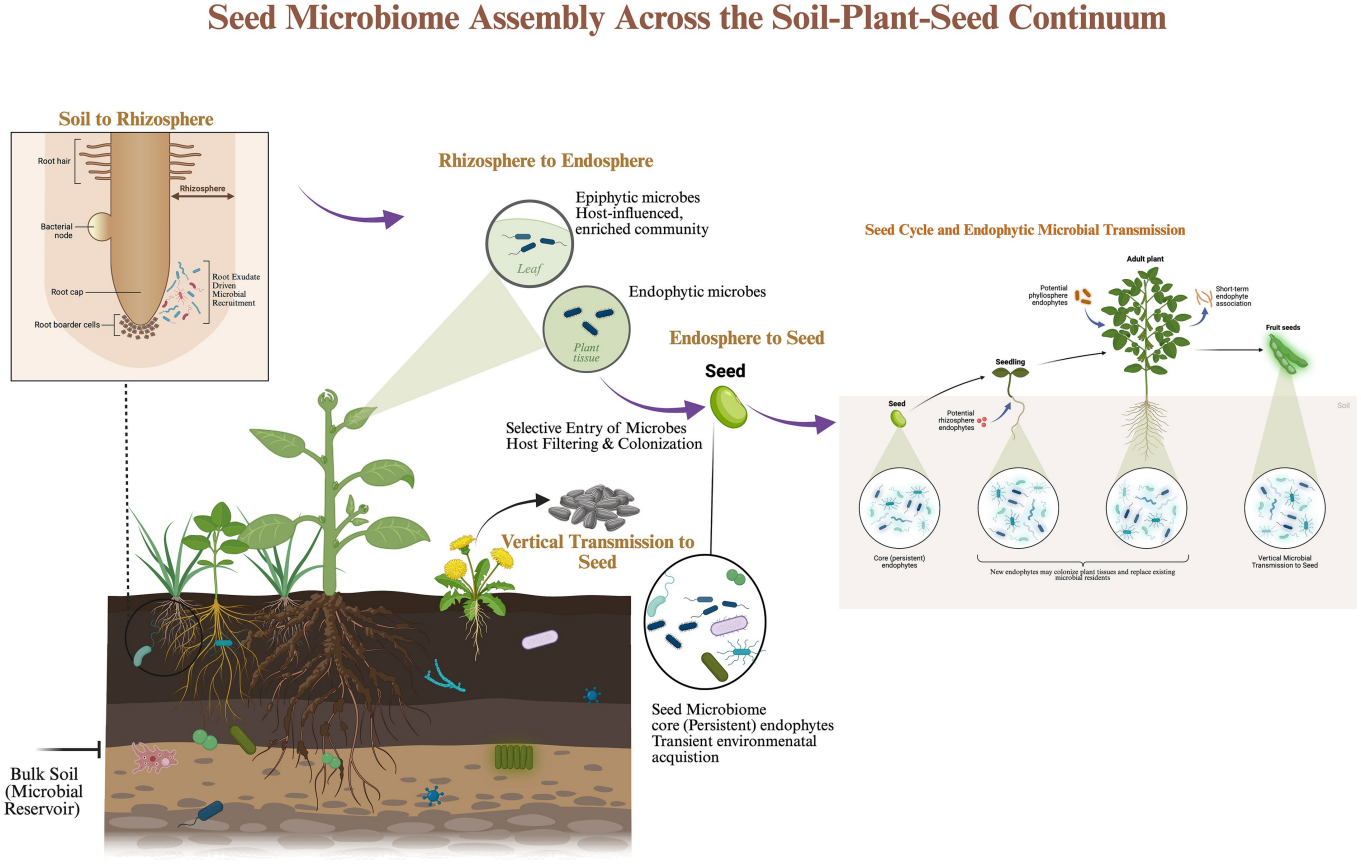
Seed microbiome assembly across the soil–plant-seed continuum. Created in BioRender, https://BioRender.com/g0w7aiw (David, 2026).

This schematic illustrates (Figure 1) the sequential pathways through which soil microorganisms contribute to seed microbiome formation across plant developmental stages. Bulk soil functions as a diverse microbial reservoir containing bacteria and fungi that are recruited to the rhizosphere by root exudate driven chemotaxis. Within the rhizosphere, a host-influenced and enriched microbial community develops, from which a subset of microorganisms enters root tissues through selective host filtering to establish the root endosphere. Endophytic microbes may subsequently move systemically through vascular tissues into aerial organs, including leaves, flowers, and developing seeds. During seed development, vertically transmitted endophytes colonize internal seed tissues and contribute to the assembly of the core seed microbiome, while additional microbes may be acquired horizontally from the surrounding environment. Upon germination, both core and transient endophytes colonize emerging seedlings, influencing microbial succession and establishing the next generation’s seed microbiome. This soil–plant-seed continuum highlights the interconnectedness of soil microbial reservoirs, plant internal microbiomes, and transgenerational microbial inheritance.

### 3.1 Vertical and horizontal transmission of seed microbiota

Seed-associated microbial communities arise through a combination of vertical (parent-to-offspring) and horizontal (environment-to-plant) transmission processes. Vertical transmission involves the transfer of microorganisms from maternal tissues to developing seeds via vascular tissues, floral organs, pollen, or ovules. Multiple studies demonstrate that vertically transmitted microbes form a conserved core microbiome that persists across generations, often dominating early seedling microbiome assembly (Berg and Raaijmakers, 2018; Johnston-Monje et al., 2021; Zeng et al., 2023). Vertically transmitted bacteria reported across crop species frequently share traits related to stress tolerance, metabolic flexibility, and host compatibility. These microbes are not passively inherited contaminants but are selectively maintained, suggesting an evolutionary advantage to host plants.

Horizontal transmission, in contrast, occurs primarily during germination and early seedling establishment, when seeds and emerging roots interact intensively with surrounding soil microbiota. Soil represents the largest source of microbial diversity; however, only a small fraction of soil microorganisms successfully colonize plant tissues. Experimental studies tracking microbial sources reveal that although soil contributes the majority of microbial taxa encountered by seedlings, strong host-mediated selection drastically reduces the number of taxa that persist internally (Rochefort et al., 2021). This demonstrates that microbial transmission is governed less by availability and more by ecological compatibility and host filtering.

Importantly, vertical and horizontal transmission are not mutually exclusive. Vertically transmitted microbes often act as priority colonizers, establishing early niches that influence subsequent recruitment of soil-derived microbes. This priority effect enables inherited microbes to shape community assembly trajectories, reinforcing functional stability during early plant development (Shade et al., 2017; Kong et al., 2019).

### 3.2 Rhizosphere endosphere reproductive tissue connectivity

Microbial movement along the soil seed continuum follows a spatially organized pathway. The rhizosphere serves as the initial recruitment interface, enriched by root exudates that stimulate microbial growth and activity. From this zone, a subset of microorganisms penetrates root epidermal and cortical tissues to establish endophytic populations. These endophytes may subsequently migrate systemically through xylem and phloem networks to aerial tissues, including stems, leaves, flowers, and developing seeds.

High-resolution microscopy and sequencing approaches provide strong evidence for this connectivity. Fluorescence *in situ* hybridization, confocal microscopy, and microdissection studies have visualized bacteria and fungi within vascular tissues, floral organs, and seed interiors, confirming active microbial movement rather than surface contamination (Frank et al., 2017; Johnston-Monje et al., 2021). Amplicon and shotgun metagenomic analyses further reveal overlapping microbial taxa across rhizosphere, endosphere, and seed compartments, supporting the existence of continuous transmission routes (Kim et al., 2022; Garrido-Sanz and Keel, 2025).

Reproductive tissues represent the most selective stage of this continuum. Only microbes possessing traits such as immune evasion, stress tolerance, and metabolic compatibility can persist within developing seeds. This strong ecological filtering results in seed microbiomes that are less diverse but functionally enriched compared to soil communities.

### 3.3 Evidence from metagenomics and imaging approaches

Advances in metagenomics have fundamentally reshaped understanding of soil seed microbial transmission. Comparative studies consistently show that seed microbiomes represent a reduced yet specialized subset of soil and plant-associated communities. Functional profiling reveals enrichment of genes associated with osmoprotection, antioxidative defense, phytohormone modulation, and nutrient mobilization traits directly relevant to seedling establishment and stress resilience (Rochefort et al., 2021; Kim et al., 2022).

Longitudinal metagenomic studies provide compelling evidence for seed-to-seed microbial continuity. In rice and bean systems, specific bacterial and fungal taxa persist across multiple plant generations despite environmental variability, with parental seeds and stem endospheres acting as dominant microbial sources (Kim et al., 2022; Sulesky-Grieb et al., 2024). Imaging-based validation strengthens these findings by confirming the physical presence of microbes along predicted transmission routes, bridging molecular inference with spatial evidence.

Together, these studies indicate that seed microbiomes are not transient assemblages but structured, heritable communities with functional significance. However, important methodological and conceptual limitations remain. Distinguishing true vertical inheritance from surface contamination or post-harvest microbial acquisition remains challenging, particularly in studies relying solely on sequencing-based inference. Variability in sterilization protocols, sequencing depth, and experimental design contributes to inconsistent evidence regarding the stability and universality of vertically transmitted core taxa. Greater integration of culture-based validation, imaging approaches, and longitudinal multi-generational studies is needed to conclusively establish mechanisms of microbial inheritance.

### 3.4 Ecological filtering under salinity stress

Salinity represents a powerful selective force shaping microbial community assembly along the soil seed continuum. In saline soils, overall microbial diversity often declines; however, salt-tolerant taxa capable of osmolyte production, ion homeostasis, and stress signaling are preferentially enriched (Abdelfadil et al., 2024). These stress-adapted microbial communities frequently dominate rhizosphere and endosphere niches of halophytes and salt-exposed crops. Seeds from salt-exposed plants are often enriched in halotolerant endophytes possessing osmoprotective and ion-regulatory traits.

Crucially, salinity influences not only soil and root microbiomes but also seed-transmitted communities. Plants exposed to saline conditions tend to pass on microbial consortia enriched in stress-mitigating functions, effectively preconditioning offspring for similar environments. This phenomenon aligns with concepts of plant soil feedback, soil memory, and microbial inheritance, where historical environmental conditions shape future plant performance through microbial legacies (Kong et al., 2019; Vannier et al., 2019).

### 3.5 Seeds as “microbial memory capsules”

Seeds can therefore be conceptualized as microbial memory capsules that store ecological information across plant generations. By selectively retaining beneficial microbes, seeds encode adaptive traits such as salinity tolerance, nutrient acquisition efficiency, and pathogen resistance. Multigenerational studies demonstrate remarkable stability of certain seed-associated microbes even under abiotic stress, underscoring their role in transgenerational resilience (Sulesky-Grieb et al., 2024; Jha et al., 2025). Persistent seed-associated taxa with stable colonization and stress adaptive traits have been shown to recur across generations, reinforcing the concept of microbial memory.

This perspective has significant implications for sustainable agriculture. Harnessing seed-associated microbiomes offers a biologically grounded strategy to enhance crop resilience while reducing dependence on chemical inputs. Integrating soil seed microbiome knowledge into breeding programs, seed treatments, and microbiome engineering approaches could enable deliberate transmission of stress-adapted microbial consortia, strengthening agroecosystem sustainability under salinity and climate change pressures.

## 4 Seed microbiome mediated salinity stress tolerance

**Figure 2:**
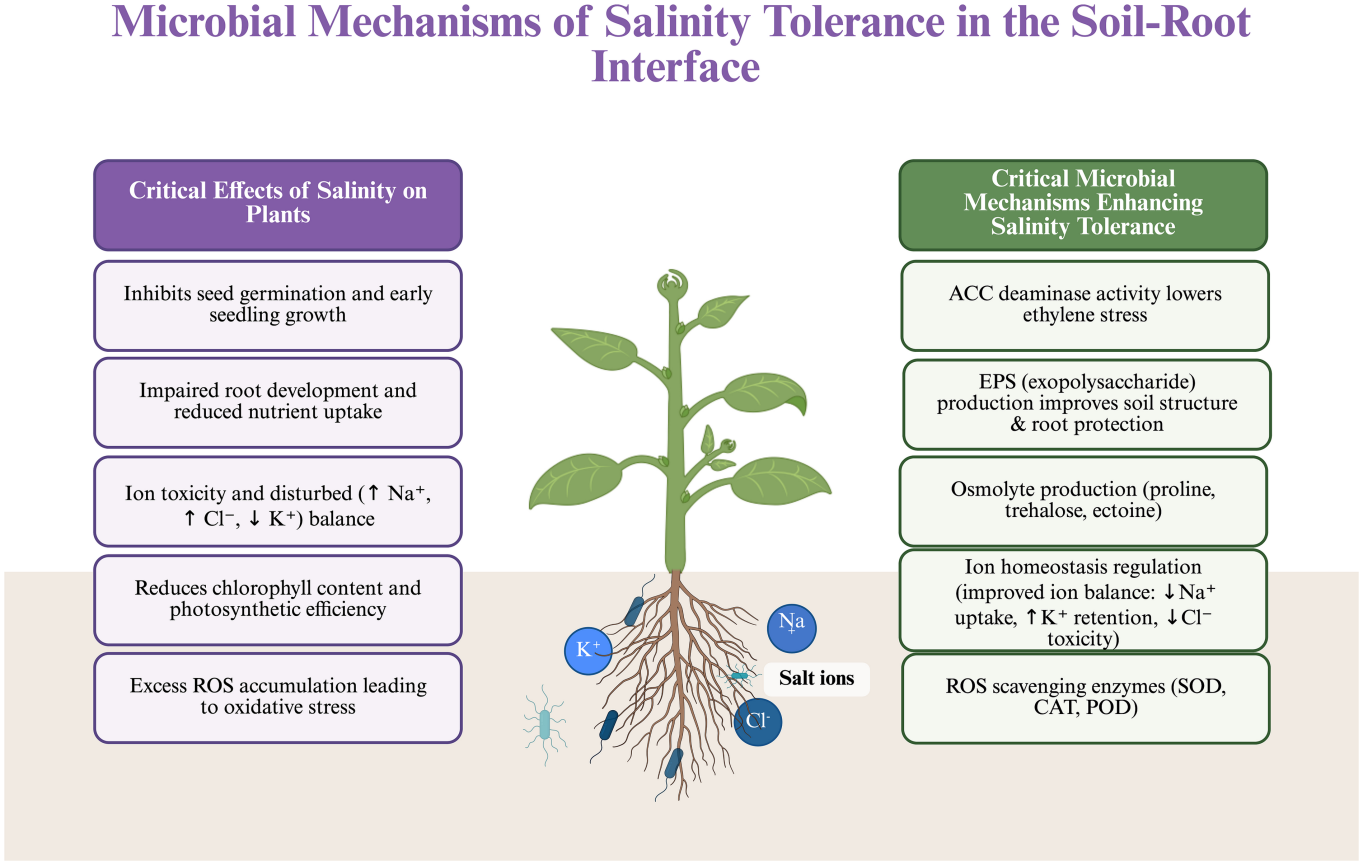
Microbial mechanisms that alleviate salinity stress at the soil-root interface.

Salinity stress disrupts plant growth by imposing osmotic imbalance, ionic toxicity, oxidative damage, and hormonal dysregulation. While plants possess intrinsic salt stress signaling pathways, accumulating evidence demonstrates that seed-associated microbiomes actively reinforce host stress tolerance through complementary physiological, molecular, and epigenetic mechanisms. Unlike transient rhizosphere associations, seed-borne microbes influence stress responses from the earliest stages of germination, thereby shaping developmental trajectories under saline conditions. The mechanistic framework underlying microbe-mediated salinity tolerance at the soil–root interface is summarized in Figure 2, and representative microbial taxa and their functional contributions are presented in Table 1.

Salinity disrupts germination, root development, ion balance, photosynthesis, and redox stability. Beneficial soil microbes counter these effects through ACC deaminase mediated ethylene reduction, EPS-driven root protection, microbial osmolyte support, improved Na^+^/K^+^ homeostasis, and enhanced antioxidant enzyme activity. Created in BioRender. David, E. (2026) https://BioRender.com/ji6qfyu.

**Table 1:** Key microbial taxa associated with soil, rhizosphere, endosphere, and seeds, highlighting their salinity-adaptation traits and functional contributions to plant tolerance under salt stress.

Functional mechanism	Representative taxa	Plant benefits under salinity	References
ACC deaminase mediated ethylene regulation	*Bacillus* spp.; *Enterobacter* spp.; *Burkholderia* spp.; *Pseudomonas* spp.	Germination; root elongation; growth enhancement; Na^+^ exclusion	Anand et al. (2021) and Azeem et al. (2022)
Osmolyte production and Osmotic balance	*Bacillus* spp.; *Microbacterium* spp.; *Halomonas* spp.; *Halobacillus* spp.; *Exiguobacterium* spp.; *Nocardiopsis* spp.; *Debaryomyces* spp.	Osmotic balance; water retention; enhanced salinity tolerance; stress resilience; root growth support	Luo et al. (2024), Liu et al. (2025), and Núñez-Cano et al. (2025)
Ion homeostasis and Na^+^/K^+^ regulation	*Halomonas* spp.; *Bacillus* spp.	Na^+^ exclusion; osmotic balance; improved growth	Azeem et al. (2022) and Hou et al. (2022)
Antioxidant enzyme induction and ROS mitigation	*Trichoderma* spp.; *Pseudomonas* spp.	Reduced ROS; root health; ISR activation	Srivastava and Srivastava (2020) and Hu et al. (2025)
Phytohormone modulation and growth regulation	*Pantoea* spp.; *Enterobacter* spp.; *Azospirillum* spp.	Root elongation; biomass accumulation; seed vigor	Johnston-Monje et al. (2021) and Degon et al. (2023)
Nitrogen fixation and nutrient mobilization	*Rhizobium* spp.; *Rhizobium melliloti*; *Kosakonia* spp.; *Azospirillum* spp.; *Paenibacillus* spp.; *Mortierella* spp.	Improved N availability; enhanced N uptake; improved P availability; nutrient mobilization	Irshad et al. (2021) and Shahid et al. (2022)
Biofilm formation and structural support	*Acinetobacter* spp.; *Microbacterium* spp.; *Bacillus* spp.	Root protection; water retention; soil structure	Patel et al. (2022) and Luo et al. (2024)
Seed persistence and early microbiome assembly	*Sphingomonas melonis*; *Alternaria* spp.; *Cladosporium* spp.; *Epicoccum* spp.; *Methylobacterium* spp.	Early seedling protection; germination support; enhances seedling vigor	Matsumoto et al. (2021) and Rétif et al. (2023)
Extreme halotolerance/soil functional stability	*Salinibacter* spp.; *Arthrobacter* spp.; *Chaetomium* spp.	Soil functional stability; survival; biocontrol + salt resilience	Krishnan et al. (2016) and Sharma and Bora (2025)

### 4.1 Physiological and molecular mechanisms

#### 4.1.1 ACC deaminase activity and ethylene homeostasis

One of the most extensively characterized microbial mechanisms conferring salinity tolerance is the production of 1-aminocyclopropane-1-carboxylate (ACC) deaminase. Under salt stress, plants accumulate ethylene, which suppresses root elongation and accelerates senescence. ACC deaminase producing bacteria cleave ACC, the immediate ethylene precursor, thereby lowering stress-induced ethylene levels and restoring root growth dynamics.

Both rhizosphere- and seed-associated ACC deaminase producing bacterial groups have been shown to mitigate salinity stress via ACC deaminase activity, resulting in improved germination, root architecture, and biomass accumulation (Anand et al., 2021; Mishra et al., 2021; Shahid et al., 2023). Importantly, seed-transmitted ACC deaminase positive microbes provide an early protective advantage, particularly during the salt-sensitive germination and seedling establishment phases.

#### 4.1.2 Osmolyte production and osmotic adjustment

Microbial modulation of compatible solutes represents another critical strategy for salinity stress mitigation. Beneficial seed-associated microbes enhance plant accumulation of osmoprotectants such as proline, trehalose, glycine betaine, and soluble sugars, which stabilize proteins, membranes, and cellular hydration under osmotic stress.

In parallel, several microbes synthesize osmolytes themselves or stimulate host biosynthetic pathways through metabolic signaling, thereby reinforcing osmotic adjustment (Arıkan et al., 2021; Gaikwad et al., 2024). Trehalose-producing microbes, in particular, play a dual role by protecting both microbial cells and host tissues, facilitating sustained colonization under saline conditions.

#### 4.1.3 Ion homeostasis and Na^+^/K^+^ balance

A defining feature of salt tolerance is the maintenance of Na^+^/K^+^ homeostasis. Excessive sodium accumulation disrupts enzymatic processes and membrane integrity, while potassium depletion compromises cellular metabolism. Seed microbiome members influence ion transport by modulating the expression and activity of plant Na^+^/H^+^ antiporters, potassium channels, and vacuolar sequestration systems.

Microbial inoculation studies demonstrate reduced Na^+^ accumulation and enhanced K^+^ retention in shoots and roots, resulting in improved photosynthetic efficiency and growth under salinity (Ali et al., 2023; Jabbar et al., 2025). These effects align with core plant salt signaling networks involving SOS pathways and calcium-mediated signaling cascades (Zhou et al., 2024), highlighting functional integration between microbial and host regulatory systems.

#### 4.1.4 Antioxidant enzyme induction and redox homeostasis

Salinity-induced oxidative stress arises from excessive generation of reactive oxygen species (ROS), leading to lipid peroxidation, DNA damage, and protein oxidation. Seed microbiome–associated bacteria and fungi enhance host antioxidant capacity by upregulating enzymatic defenses, including superoxide dismutase, catalase, peroxidase, and ascorbate–glutathione cycle enzymes.

Metabolite-mediated signaling between microbes and plants further fine-tunes redox homeostasis, preventing oxidative damage while preserving ROS signaling functions essential for stress adaptation (Khawula et al., 2025; Sharma et al., 2025). These antioxidant responses are particularly critical during early seedling development, when endogenous defense systems are not fully established.

### 4.2 Hormonal and epigenetic modulation by seed microbiomes

#### 4.2.1 Phytohormone regulation and growth stress balance

Beyond stress alleviation, seed microbiomes actively regulate phytohormone networks, integrating growth promotion with stress tolerance. Many seed-associated microbes synthesize or modulate auxins, gibberellins, cytokinins, and abscisic acid, thereby influencing root system architecture, stomatal behavior, and resource allocation under salinity.

Auxin-producing microbes promote lateral root formation and root hair development, enhancing water and nutrient uptake in saline soils. Cytokinin modulation delays stress-induced senescence, while microbial regulation of ABA signaling fine-tunes stomatal closure and osmotic balance (Singh et al., 2023; Li et al., 2025). This hormonal crosstalk ensures that stress mitigation does not occur at the expense of growth and yield.

#### 4.2.2 Stress memory, epigenetic regulation, and transgenerational priming

Emerging evidence suggests that seed microbiomes contribute to stress memory and transgenerational priming. Exposure of parent plants to salinity alters seed-associated microbial composition, enriching taxa capable of inducing stress-responsive gene expression and chromatin modifications in progeny. These effects intersect with host epigenetic mechanisms, including DNA methylation, histone modifications, and small RNA signaling.

Seed bio-priming studies demonstrate that microbiome-mediated priming enhances tolerance not only during immediate germination but also throughout later developmental stages (Chakraborti et al., 2022; Srivastava et al., 2024). Such primed states persist across generations, suggesting that seed microbiomes function as carriers of ecological information, reinforcing adaptive phenotypes under recurring salinity stress (Hasanović et al., 2025). Seed-transmitted microbes possessing stress inducible regulatory and signaling traits have been implicated in inducing heritable stress-responsive transcriptional and epigenetic states in progeny. However, distinguishing true microbial inheritance from environmental re-acquisition or host-driven epigenetic memory remains methodologically challenging. Much of the current evidence derives from controlled bio-priming experiments or short-term generational studies, and causal mechanisms linking specific microbial taxa to stable epigenetic modifications require further longitudinal validation. Thus, microbial-mediated stress memory should be considered a promising but still emerging framework.

These findings establish seed microbiomes as active regulators of plant salinity tolerance, operating through interconnected physiological, molecular, hormonal, and epigenetic pathways. By aligning microbial functions with plant salt stress signaling networks, seed-associated microbes enhance resilience from germination onward. This mechanistic understanding provides a foundation for microbiome informed seed technologies and sustainable strategies to mitigate salinity stress in agroecosystems.

## 5 Soil microbiome influence on seed quality and plant health

The soil microbiome exerts a decisive influence on seed quality and subsequent plant health by shaping early developmental processes, regulating biotic interactions, and stabilizing productivity under environmental stress. Through plant soil feedback mechanisms, soil microorganisms indirectly and directly affect seed vigor, microbial inheritance, and host resilience, thereby linking soil health to crop performance across generations.

### 5.1 Germination vigor and seedling establishment

Seed germination and early seedling establishment represent critical bottlenecks in plant life cycles, particularly under stress-prone environments. Soil microbial communities influence these stages by modulating nutrient availability, phytohormone balance, and microbial colonization dynamics at the seed soil interface. Beneficial soil microorganisms enhance enzymatic mobilization of seed reserves, stimulate radicle emergence, and promote uniform seedling establishment (Vincze et al., 2024; Adeboye et al., 2025).

Recent evidence from successional ecology demonstrates that plant soil microbe feedbacks regulate seedling recruitment and establishment, with soil microbial legacies exerting stage-specific effects on plant performance (Liu and Zhao, 2023). Such feedbacks are particularly relevant in saline and degraded soils, where microbial conditioning of the soil environment determines seedling survival probability.

Seed-associated microbes recruited from soil further reinforce germination vigor by preconditioning seeds with growth-promoting metabolites and stress-buffering functions. Microbial seed inoculation and bio-priming approaches have consistently improved emergence rates and early biomass accumulation under controlled and field conditions, with reported increases in germination percentage and seedling biomass across saline soils (O’Callaghan, 2016; Chakraborti et al., 2022). Together, these findings indicate that soil microbiome composition influences seed-associated microbial assembly and metabolic conditioning, thereby directly shaping germination vigor and early seedling establishment under stress.

### 5.2 Disease suppression and pathogen exclusion

One of the most robust contributions of soil microbiomes to plant health is their role in disease suppressiveness. Healthy soils harbor diverse microbial consortia capable of inhibiting pathogens through competition for nutrients and niches, antibiosis, parasitism, and induction of plant systemic resistance. These mechanisms operate at both the rhizosphere and seed levels, creating multilayered barriers against infection.

Seed-recruited microbiomes originating from disease-suppressive soils have been shown to protect seedlings by disrupting pathogen recognition and colonization processes. For example, seed-associated microbial assemblages can alter chemotactic responses of soil-borne pathogens, thereby preventing infection during early development (Jack and Nelson, 2018). Such protective effects highlight the importance of microbial inheritance in plant defense strategies.

Comprehensive analyses of plant microbe interactions across diverse cropping systems confirm that soil microbiome composition strongly predicts disease resilience, particularly under stress conditions that otherwise predispose plants to infection (Noman et al., 2021; Amoo et al., 2023). Soil health management practices that enhance microbial diversity and functional redundancy therefore indirectly improve seed health and reduce disease incidence in subsequent plant generations (Babu et al., 2024; Priyadarshini et al., 2025). Disease-suppressive seed microbiomes commonly include antagonistic taxa such as *Pseudomonas*, *Bacillus*, *Streptomyces*, and *Trichoderma*, which inhibit pathogens through competition and antibiosis. These observations reinforce that shifts in soil microbial community structure cascade into seed-associated microbiomes, strengthening pathogen exclusion mechanisms from the earliest developmental stages.

### 5.3 Yield stability under stress conditions

Yield stability under abiotic stress is increasingly recognized as an emergent property of soil plant microbe interactions. Beneficial soil microbiomes enhance nutrient acquisition efficiency, improve nitrogen use recovery, and stabilize physiological processes under salinity, drought, and temperature extremes. These effects translate into consistent yield performance across variable environments.

Field-based studies across stress affected agroecosystems demonstrate that plant growth promoting bacteria improve grain yield, protein content, and nutrient recovery, particularly in stress-affected soils (Martins et al., 2018). When such microbes are transmitted to seeds or influence seed-associated microbiomes, their benefits extend beyond a single growing season, reinforcing resilience at the population level. Thus, long-term soil microbiome composition not only stabilizes soil functions but also conditions seed-associated communities that contribute to sustained yield performance across environmental gradients.

Long-term soil microbiome stewardship further contributes to yield stability by maintaining soil structure, organic matter turnover, and nutrient cycling capacity. Systematic reviews spanning multiple decades confirm that soils with high microbial functional diversity exhibit greater buffering capacity against climate-induced stressors (Etesami, 2024). In this context, seed quality emerges not merely as a genetic attribute but as a biologically conditioned trait shaped by soil microbial history. Collectively, these insights position the soil microbiome as a central determinant of seed quality, plant health, and yield stability. By governing germination success, suppressing disease, and enhancing stress resilience, soil microorganisms create a functional bridge between soil sustainability and agricultural productivity. Leveraging this soil seed plant continuum represents a critical pathway toward resilient cropping systems and sustainable intensification under global environmental change.

## 6 Modern tools and technologies to harness soil seed microbiomes

The increasing recognition of soil and seed microbiomes as functional determinants of plant health and stress resilience has accelerated the development of advanced tools to characterize, design, and deploy beneficial microbial communities. Modern technologies spanning multi-omics, microbial engineering, seed-based delivery systems, and artificial intelligence are transforming microbiome research from descriptive ecology toward predictive and application-oriented science.

### 6.1 Multi-omics approaches to decode soil seed microbiome function

High-throughput metagenomics, metatranscriptomics, metaproteomics, and metabolomics have become indispensable for elucidating the taxonomic composition and functional potential of soil and seed-associated microbiomes. Shotgun metagenomics enables strain-level resolution of microbial communities, revealing functional genes associated with nutrient cycling, stress tolerance, siderophore production, and phytohormone biosynthesis (Nam et al., 2023; Galanova et al., 2025).

Metatranscriptomic profiling further distinguishes metabolically active microbial populations, capturing context-dependent gene expression during seed germination, root colonization, and stress exposure. Such approaches have proven particularly valuable in plant disease management and stress biology by identifying microbial pathways responsive to host signals and environmental perturbations (Vannier et al., 2023; Peng et al., 2024).

Integration of omics datasets has revealed functional connectivity between rhizosphere microbiomes and host gene networks, demonstrating coordinated regulation of nutrient uptake, immunity, and stress signaling (Fadiji et al., 2023). Importantly, seed-focused omics studies highlight that microbial functional traits linked to vigor and yield are already imprinted at the seed stage, reinforcing the soil seed microbiome continuum (Cummane et al., 2025).

### 6.2 Culturomics and recovery of functional microbial diversity

While omics approaches provide comprehensive community profiles, culturomics bridges the gap between sequence data and practical application by enabling the isolation and functional validation of previously unculturable microbes. Recent advances in high-throughput culturing, microfluidics, and customized growth media have significantly expanded the cultivable fraction of soil and seed microbiomes (Sarhan et al., 2019).

Culturomics-guided strategies have facilitated the recovery of stress-adapted and keystone taxa capable of producing siderophores, exopolysaccharides, osmoprotectants, and antimicrobial compounds. These isolates form the foundation for developing microbial inoculants tailored to specific soil constraints, including salinity and nutrient limitation (Santoyo et al., 2021; AbuQamar et al., 2024; Loiko and Islam, 2024; Renganathan et al., 2025).

By integrating culturomics with metagenomic insights, researchers can prioritize functionally relevant microbes rather than relying solely on taxonomic abundance, enhancing the reliability and reproducibility of microbiome-based interventions.

### 6.3 Synthetic microbial consortia and microbiome engineering

Moving beyond single-strain inoculants, synthetic microbial consortia represent a next-generation approach to harness microbiome functions. Carefully designed consortia exploit functional complementarity among microbial members, improving stability, resilience, and efficacy under field conditions.

Consortia-based strategies enable simultaneous delivery of multiple traits, such as nutrient solubilization, pathogen suppression, and stress mitigation. Advances in top-down and bottom-up microbiome engineering have facilitated rational assembly of microbial communities guided by ecological principles, network analysis, and functional redundancy (Lyu et al., 2024).

Such engineered consortia are particularly promising for seed and soil applications, where microbial persistence and compatibility with native communities are critical determinants of success (Herath Dissanayakalage et al., 2025; Portal-Gonzalez et al., 2025; Sharma and Bora, 2025). Importantly, synthetic microbiomes also offer platforms for studying emergent properties of plant microbe interactions under controlled yet ecologically relevant conditions. In the context of salinity stress, synthetic consortia can be specifically designed to include halotolerant and seed-transmissible members, ensuring persistence across plant developmental stages and enhancing early-life resilience under saline conditions.

### 6.4 Seed coating, microbial priming, and targeted delivery systems

Seeds represent an efficient and scalable vehicle for microbiome deployment. Seed coating, bio-priming, and encapsulation technologies enable targeted delivery of beneficial microbes at the earliest stages of plant development, ensuring immediate functional engagement with the host.

Modern seed treatments incorporate microbial consortia, biostimulants, and protective polymers to enhance microbial survival, adhesion, and colonization efficiency. Such approaches improve germination performance, disease resistance, and stress tolerance while minimizing the need for repeated soil applications (O’Callaghan, 2016; Cardarelli et al., 2022; Garg et al., 2024).

Microbial seed priming further induces physiological and molecular preparedness in plants, activating stress-responsive pathways and reinforcing stress memory mechanisms. These technologies align well with sustainable agriculture goals by reducing chemical inputs and enhancing biological resilience (Cummane et al., 2025).

### 6.5 Precision soil management and AI-driven microbiome design

The complexity and context dependency of soil seed microbiomes necessitate data-driven and predictive frameworks. Artificial intelligence (AI) and machine learning models are increasingly applied to integrate multi-omics data, soil physicochemical parameters, climate variables, and agronomic practices.

AI-driven microbiome analysis enables identification of key functional predictors of plant performance and stress tolerance, supporting rational selection of microbial traits and consortia (Topçuoğlu et al., 2020). Such predictive frameworks are particularly valuable for identifying microbial traits associated with seed transmission and salinity adaptation, enabling rational design of seed-applied consortia tailored to salt-affected agroecosystems. Deep learning approaches further facilitate microbiome design by predicting microbial interactions, community stability, and host compatibility across environments.

Precision soil management systems that combine microbiome analytics with real-time soil monitoring offer new opportunities to optimize microbial interventions at field scale. Such integrative approaches align with emerging green technology frameworks aimed at climate-resilient and regenerative agriculture (Schmidt et al., 2019; Trivedi et al., 2020).

Despite these technological advances, significant challenges remain in translating microbiome-based innovations from controlled environments to field-scale application. Reproducibility of microbial inoculants across diverse soils, climatic conditions, and cropping systems remains inconsistent, and microbial persistence is often influenced by native community resistance and environmental variability. Furthermore, regulatory frameworks governing microbial products vary across regions, potentially limiting large-scale deployment. Addressing these constraints through standardized validation protocols and long-term field trials is essential for realizing the full potential of soil seed microbiome technologies.

## 7 Implications for sustainable agriculture and soil sustainability

Harnessing soil seed microbiomes offers a transformative pathway to reconcile agricultural productivity with environmental sustainability. By embedding microbial functions into seed and soil management strategies, agriculture can transition from input-intensive practices toward biologically driven, resilient systems that sustain soil health, crop performance, and ecosystem services.

### 7.1 Reduced dependence on chemical inputs

One of the most immediate implications of soil seed microbiome integration is the reduction of synthetic fertilizers and pesticides. Beneficial soil microorganisms enhance nutrient availability through biological nitrogen fixation, phosphorus solubilization, and micronutrient mobilization, thereby improving nutrient use efficiency and reducing fertilizer losses. When these functions are transmitted through seeds or reinforced during early plant development, nutrient acquisition becomes more synchronized with crop demand.

Similarly, disease-suppressive soil microbiomes and seed-associated protective consortia reduce reliance on chemical pesticides by limiting pathogen establishment and enhancing plant immune competence. Sustainable agricultural frameworks increasingly recognize that soil biological fertility can substitute for chemical inputs, lowering production costs and minimizing environmental contamination (De Corato et al., 2024; Țopa et al., 2025). This shift is particularly relevant in saline and degraded soils, where chemical inputs often exacerbate soil structural decline and microbial imbalance. However, field-scale adoption depends on consistent performance across soil types and seasons, and long-term validation trials are required to quantify reductions in fertilizer input, pesticide use, and yield variability under diverse agroecological conditions.

### 7.2 Enhanced crop resilience and productivity under stress

Soil seed microbiome-based strategies directly enhance crop resilience to abiotic and biotic stresses, including salinity, drought, and nutrient limitation. By improving early seedling establishment, regulating stress-responsive pathways, and stabilizing plant soil feedbacks, microbiome-informed systems support consistent productivity across variable climatic conditions.

Recent assessments of climate-resilient agriculture emphasize that soil health centered approaches outperform conventional practices in maintaining yield stability under stress scenarios (Kabato et al., 2025). The soil seed microbiome continuum strengthens this resilience by ensuring that beneficial microbial functions are present from germination onward, reducing vulnerability during critical developmental windows.

Field-scale evaluations further demonstrate that biologically managed soils exhibit improved aggregation, water retention, and carbon stabilization, all of which indirectly contribute to sustained crop productivity (Çakmakçi et al., 2025). Thus, microbiome-based interventions not only enhance short-term yields but also reinforce the long-term productive capacity of agricultural soils. Indicators such as yield stability across stress years, nutrient use efficiency metrics, soil organic carbon accumulation, and reduced disease incidence provide measurable benchmarks for evaluating microbiome-based success at farm scale.

### 7.3 Contribution to the United Nations sustainable development goals

The integration of soil and seed microbiomes is strongly aligned with several United Nations Sustainable Development Goals (SDGs), underscoring their relevance in advancing sustainable and climate-resilient agricultural systems. By enhancing nutrient-use efficiency, stabilizing yields, and improving crop resilience in marginal and saline soils, microbiome-based agricultural strategies directly contribute to SDG 2 (Zero Hunger) through sustainable intensification and improved food security, particularly in stress-prone agroecosystems (Hiywotu, 2025). Simultaneously, the reduced reliance on synthetic fertilizers and pesticides achieved through biologically mediated nutrient cycling and disease suppression supports SDG 12 (Responsible Consumption and Production) by lowering input demands and minimizing the environmental footprint of agricultural production systems.

Healthy, microbially active soils also play a critical role in climate mitigation and adaptation, thereby advancing SDG 13 (Climate Action). Through enhanced soil carbon sequestration, improved soil structure, and increased buffering capacity against climatic extremes, soil microbiomes strengthen agroecosystem resilience under increasing climate variability (Lal et al., 2021; Kabato et al., 2025). Furthermore, the conservation and targeted management of soil and seed-associated microbial diversity contribute to SDG 15 (Life on Land) by promoting soil biodiversity, facilitating land restoration, and supporting sustainable land-use practices. From a global soil governance perspective, soil microorganisms are increasingly recognized as foundational biological assets that link soil health, ecosystem resilience, and sustainable development, positioning microbiome stewardship as a strategic lever for achieving multiple SDG targets simultaneously (Lal et al., 2021; Çakmakçi et al., 2025). Nevertheless, achieving these outcomes requires overcoming barriers including variability in microbial performance, farmer adoption constraints, cost of inoculant development, and the need for regulatory harmonization across regions. Trade-offs between short-term yield optimization and long-term soil biological investment must also be carefully managed to ensure sustained benefits.

### 7.4 Nature-based solutions for soil restoration

Soil seed microbiome strategies represent a powerful class of nature-based solutions (NbS) for restoring degraded and saline soils. Unlike mechanical or chemical remediation approaches, microbiome-driven restoration leverages ecological processes such as microbial succession, organic matter turnover, and plant microbe co-adaptation.

Recent synthesis studies highlight that restoring soil biological function accelerates recovery of soil structure, nutrient cycling, and plant productivity, particularly when combined with sustainable farming practices such as reduced tillage, organic amendments, and diversified cropping systems (Mrunalini et al., 2022; Neuenkamp et al., 2024).

Embedding beneficial microbes into seeds further enhances the scalability of NbS by ensuring consistent microbial establishment across landscapes. Such approaches are well suited for circular bioeconomy models, where soil restoration, waste recycling, and sustainable production are integrated into a single framework. However, scalability depends on site-specific microbial compatibility, climate conditions, and integration with agronomic practices, underscoring the importance of adaptive management strategies.

## 8 Future perspectives and knowledge gaps

Despite rapid advances in soil and seed microbiome research, significant knowledge gaps remain that currently limit the translation of microbiome science into scalable and reliable agricultural solutions. One of the most pressing needs is the establishment of long-term, multi-season field studies that evaluate microbiome-mediated benefits under realistic agronomic and environmental conditions. While controlled experiments and short-term trials have convincingly demonstrated the potential of beneficial microbes to enhance salinity tolerance and crop performance, their stability, persistence, and functional consistency across years, soil types, and climatic regimes remain insufficiently understood. Long-term datasets are essential to assess microbial resilience, ecological feedbacks, and unintended consequences under continuous cultivation and climate variability. Among these gaps, the most limiting for salinity-focused applications are the lack of long-term field validation under salt-affected conditions and insufficient understanding of microbial persistence across successive plant generations in saline soils.

Another major challenge lies in the crop-specific and region-specific nature of soil seed microbiomes. Microbial assemblages and their functional traits are strongly shaped by host genotype, soil physicochemical properties, land-use history, and local climate. Consequently, universal microbial solutions are unlikely to be effective across diverse agroecosystems. This challenge is particularly acute in salt-affected regions, where soil salinity gradients, irrigation practices, and seasonal variability create highly dynamic microbial selection pressures that complicate the design of stable seed-transmitted consortia. Future research must move toward context-aware microbiome design, integrating crop genetics, soil health indicators, and regional environmental constraints to develop tailored microbial consortia and management strategies. Advances in comparative metagenomics, pan-microbiome analysis, and host microbe co-adaptation studies will be critical for identifying core versus context-dependent microbial functions relevant to salinity tolerance and sustainability.

Technical standardization also remains a critical bottleneck. Variability in sampling strategies, surface sterilization protocols, sequencing depth, and bioinformatic pipelines can lead to inconsistent detection of seed-associated microbes, complicating reproducibility across studies. Contamination control during seed microbiome analysis and the distinction between true vertical inheritance and environmental re-acquisition require more rigorous methodological harmonization. Establishing standardized experimental protocols and reporting frameworks will be essential to strengthen comparability and translational reliability.

In parallel, regulatory, scalability, and adoption challenges must be addressed to enable widespread implementation of microbiome-based interventions. Current regulatory frameworks for microbial inoculants and seed treatments are often fragmented, regionally inconsistent, or inadequately equipped to evaluate complex microbial consortia. Moreover, large-scale production, formulation stability, shelf life, and field performance of microbial products remain technical bottlenecks. Bridging the gap between laboratory innovation and farm-level adoption will require coordinated efforts involving microbiologists, agronomists, industry stakeholders, policymakers, and farmers. Socioeconomic considerations, cost benefit analyses, and farmer-centric validation trials should be integrated into future research agendas to ensure that microbiome technologies are both scientifically robust and practically viable. Prioritizing standardized validation pipelines, multi-site salinity trials, and reproducible seed microbiome characterization will determine the pace at which microbiome-based strategies transition from experimental promise to agronomic reliability.

## 9 Conclusion

This Review highlights the emerging view of soil and seed microbiomes as interconnected and functionally integrated systems, rather than isolated microbial compartments. The soil seed microbiome continuum represents a novel, integrative framework for understanding salinity resilience through which soil health, plant development, and stress resilience are linked across plant life cycles and generations. By shaping early microbial colonization, influencing physiological and molecular stress responses, and transmitting adaptive traits to subsequent plant generations, seed-associated microbes act as strategic conduits between soil ecosystems and crop performance under saline stress conditions.

Microbiome-based agriculture, particularly when embedded within sustainable soil management frameworks, represents a biologically grounded strategy for addressing salinity stress, reducing dependence on chemical inputs, and enhancing crop resilience under climate change. Harnessing beneficial soil and seed microbiomes offers a nature-based, resource-efficient pathway to improve productivity while safeguarding soil biodiversity and ecosystem services. However, realizing this potential will require moving beyond descriptive studies toward predictive, mechanism-driven, and systems-level approaches. Priority should be given to long-term field validation in salt-affected systems, standardized seed microbiome characterization protocols, and rigorous evaluation of transgenerational microbial effects.

Looking forward, the successful integration of microbiome science into mainstream agriculture will depend on interdisciplinary research, combining soil ecology, plant physiology, genomics, data science, and socioeconomics, alongside supportive policy frameworks that promote innovation while ensuring biosafety and sustainability. By aligning scientific discovery with agronomic practice and global sustainability goals, soil seed microbiome stewardship can play a pivotal role in shaping resilient food systems and sustainable agriculture for the future.

## References

[ref1] Abdelfadil M. R. Patz S. Kolb S. Ruppel S. (2024). Unveiling the influence of salinity on bacterial microbiome assembly of halophytes and crops. Environ. Microbiome 19:49-. doi: 10.1186/S40793-024-00592-3/FIGURES/4 39026296 PMC11256479

[ref2] AbuQamar S. F. El-Saadony M. T. Saad A. M. Desoky E. S. M. Elrys A. S. El-Mageed T. A. A. . (2024). Halotolerant plant growth-promoting rhizobacteria improve soil fertility and plant salinity tolerance for sustainable agriculture—a review. Plant Stress 12:100482. doi: 10.1016/J.STRESS.2024.100482

[ref3] Adeboye K. A. Fayose C. A. Ayangbenro A. S. Oyetunde O. A. Awopegba T. M. Adeleke B. S. . (2025). Harnessing microbial communities to enhance seed germination: a review of opportunities and challenges. Discover Agriculture 3:258-. doi: 10.1007/S44279-025-00437-8

[ref4] Ali B. Hafeez A. Afridi M. S. Javed M. A. Sumaira N. Suleman F. . (2023). Bacterial-mediated salinity stress tolerance in maize (*Zea mays* L.): a fortunate way toward sustainable agriculture. ACS Omega 8, 20471–20487. doi: 10.1021/ACSOMEGA.3C00723/ASSET/IMAGES/LARGE/AO3C00723_0011.JPEG 37332827 PMC10275368

[ref5] Amoo A. E. Olanrewaju O. S. Babalola O. O. Ajilogba C. F. Chukwuneme C. F. Ojuederie O. B. . (2023). The functionality of plant-microbe interactions in disease suppression. J. King Saud Univ. Sci. 35:102893. doi: 10.1016/J.JKSUS.2023.102893

[ref6] Anand G. Bhattacharjee A. Shrivas V. L. Dubey S. Sharma S. (2021). ACC deaminase positive Enterobacter-mediated mitigation of salinity stress, and plant growth promotion of *Cajanus cajan*: a lab to field study. Physiol. Mol. Biol. Plants 27, 1547–1557. doi: 10.1007/S12298-021-01031-0, 34366596 PMC8295421

[ref7] Arıkan Ş. İpek M. Pırlak L. Ahmet E. (2021). “Physiological and molecular mechanisms in improving salinity stress tolerance by beneficial microorganisms in plants,” in Microbial Management of Plant Stresses: Current Trends, Application and Challenges, Cambridge, UK: Woodhead, 13–43.

[ref8] Azeem M. A. Shah F. H. Ullah A. Ali K. Jones D. A. Khan M. E. H. . (2022). Biochemical characterization of halotolerant *Bacillus safensis* PM22 and its potential to enhance growth of maize under salinity stress. Plants 11:1721. doi: 10.3390/PLANTS11131721/S1 35807673 PMC9268828

[ref9] Babu S. Prasad S. Vaishnavi D. S. Harikrishna K. Reddy K. V. (2024). “Soil microorganisms and mycotoxins,” in Soil Microbiome in Green Technology Sustainability, Cham, Switzerland: Springer Nature Switzerland AG, 339–428.

[ref10] Bargaz A. Lyamlouli K. Chtouki M. Zeroual Y. Dhiba D. (2018). Soil microbial resources for improving fertilizers efficiency in an integrated plant nutrient management system. Front. Microbiol. 9:364232. doi: 10.3389/FMICB.2018.01606/XML PMC6079243 30108553

[ref11] Berg G. Raaijmakers J. M. (2018). Saving seed microbiomes. ISME J. 12, 1167–1170. doi: 10.1038/S41396-017-0028-2, 29335636 PMC5931960

[ref12] Çakmakçi R. Çakmakçi S. Çakmakçi M. F. (2025). Principles of environmentally sustainable agriculture for building resilient and resource-efficient food systems. Turk. J. Biol. 49, 550–584. doi: 10.55730/1300-0152.2764, 41246231 PMC12614370

[ref13] Cardarelli M. Woo S. L. Rouphael Y. Colla G. (2022). Seed treatments with microorganisms can have a biostimulant effect by influencing germination and seedling growth of crops. Plants 11:259. doi: 10.3390/PLANTS11030259, 35161239 PMC8838022

[ref14] Chakraborti S. Bera K. Sadhukhan S. Dutta P. (2022). Bio-priming of seeds: plant stress management and its underlying cellular, biochemical and molecular mechanisms. Plant Stress 3:100052. doi: 10.1016/J.STRESS.2021.100052

[ref15] Chen Q. Song Y. An Y. Lu Y. Zhong G. (2024). Soil microorganisms: their role in enhancing crop nutrition and health. Diversity 16:734. doi: 10.3390/D16120734

[ref16] Cummane J. Thomas W. J. W. Lee M. Sayari M. Edwards D. Batley J. . (2025). Omics for improving seed quality and yield. Seeds 4:49. doi: 10.3390/SEEDS4040049

[ref1000] David E. (2026). Available at: https://BioRender.com/g0w7aiw

[ref17] De Corato U. Viola E. Keswani C. Minkina T. (2024). Impact of the sustainable agricultural practices for governing soil health from the perspective of a rising Agri-based circular bioeconomy. Appl. Soil Ecol. 194:105199. doi: 10.1016/J.APSOIL.2023.105199

[ref18] Degon Z. Dixon S. Rahmatallah Y. Galloway M. Gulutzo S. Price H. . (2023). *Azospirillum brasilense* improves rice growth under salt stress by regulating the expression of key genes involved in salt stress response, abscisic acid signaling, and nutrient transport, among others. Front. Agron. 5:1216503. doi: 10.3389/FAGRO.2023.1216503 /BIBTEX, 38223701 PMC10785826

[ref19] del Rodríguez Río Á. Scheu S. Rillig M. C. (2025). Soil microbial responses to multiple global change factors as assessed by metagenomics. Nat. Commun. 16:5058. doi: 10.1038/s41467-025-60390-4, 40447574 PMC12125317

[ref20] Etesami H. (2024). Enhancing soil microbiome resilience: the mitigating role of silicon against environmental stresses. Front. Agron. 6:1465165. doi: 10.3389/FAGRO.2024.1465165 /XML

[ref21] Fadiji A. E. Yadav A. N. Santoyo G. Babalola O. O. (2023). Understanding the plant-microbe interactions in environments exposed to abiotic stresses: an overview. Microbiol. Res. 271:127368. doi: 10.1016/J.MICRES.2023.127368, 36965460

[ref22] Frank A. C. Guzmán J. P. S. Shay J. E. (2017). Transmission of bacterial endophytes. Microorganisms 5:70. doi: 10.3390/MICROORGANISMS5040070, 29125552 PMC5748579

[ref23] Futa B. Gmitrowicz-Iwan J. Skersienė A. Šlepetienė A. Parašotas I. (2024). Innovative soil management strategies for sustainable agriculture. Sustainability 2024, Vol. 16, Page:9481 16,:9481. doi: 10.3390/SU16219481

[ref24] Gaikwad A. S. Bhakare B. Kamble B. Thakare R. Durgude A. (2024). Soil microbiome: applications and mechanisms for salinity stress mitigation in plant and soil ecology: a review. Int. J. Adv. Biochem. Res. 8, 923–946. doi: 10.33545/26174693.2024.V8.I3K.875

[ref25] Galanova O. O. Mitkin N. A. Danilova A. A. Pavshintsev V. V. Tsybizov D. A. Zakharenko A. M. . (2025). Assessment of soil health through metagenomic analysis of bacterial diversity in Russian black soil. Microorganisms 13:854. doi: 10.3390/MICROORGANISMS13040854/S1 40284690 PMC12029357

[ref26] Garg S. Nain P. Kumar A. Joshi S. Punetha H. Sharma P. K. . (2024). Next generation plant biostimulants & genome sequencing strategies for sustainable agriculture development. Front. Microbiol. 15:1439561. doi: 10.3389/FMICB.2024.1439561/XML 39104588 PMC11299335

[ref27] Garrido-Sanz D. Keel C. (2025). Seed-borne bacteria drive wheat rhizosphere microbiome assembly via niche partitioning and facilitation. Nat. Microbiol. 10, 1130–1144. doi: 10.1038/s41564-025-01973-1, 40140705 PMC12055584

[ref28] Hasanović M. Durmić-Pašić A. Karalija E. (2025). Seed priming beyond stress adaptation: broadening the agronomic horizon. Agronomy 15:1829. doi: 10.3390/AGRONOMY15081829

[ref29] Herath Dissanayakalage S. S. Kaur J. Li T. Dimech A. M. Sawbridge T. I. (2025). Seed-derived synthetic microbial communities (SynComs) from Medicago wild relatives modulate early plant microbiome assembly and phenotypic traits in lucerne (*Medicago sativa* L.). Microorganisms 13:2114. doi: 10.3390/MICROORGANISMS13092114/S1, 41011445 PMC12472412

[ref30] Hiywotu A. M. (2025). Advancing sustainable agriculture for goal 2: zero hunger - a comprehensive overview of practices, policies, and technologies. Agroecol. Sustain. Food Syst. 49, 1027–1055. doi: 10.1080/21683565.2025.2451344

[ref31] Hou Y. Zeng W. Ao C. Luo Y. Wang Z. Hou M. . (2022). *Bacillus atrophaeus* WZYH01 and Planococcus soli WZYH02 improve salt tolerance of maize (*Zea mays* L.) in saline soil. Front. Plant Sci. 13:891372. doi: 10.3389/FPLS.2022.891372/BIBTEX 35599881 PMC9121094

[ref32] Hu H. Geng S. Zhu Y. He X. Pan X. Yang M. (2025). Seed-borne endophytes and their host effects. Microorganisms 13:842. doi: 10.3390/MICROORGANISMS13040842, 40284678 PMC12029701

[ref33] Iqbal S. Begum F. Nguchu B. A. Claver U. P. Shaw P. (2025). The invisible architects: microbial communities and their transformative role in soil health and global climate changes. Environ. Microbiome 20:36. doi: 10.1186/S40793-025-00694-6, 40133952 PMC11938724

[ref34] Irshad A. Rehman R. N. U. Abrar M. M. Saeed Q. Sharif R. Hu T. (2021). Contribution of Rhizobium–legume symbiosis in salt stress tolerance in *Medicago truncatula* evaluated through photosynthesis, antioxidant enzymes, and compatible solutes accumulation. Sustainability 13:3369. doi: 10.3390/SU13063369

[ref35] Islam W. Zeng F. Alotaibi M. O. Khan K. A. (2024). Unlocking the potential of soil microbes for sustainable desertification management. Earth-Sci. Rev. 252:104738. doi: 10.1016/J.EARSCIREV.2024.104738

[ref36] Jabbar A. Zuan A. T. K. Mahmood A. (2025). “The role of microbiome in crop salinity tolerance,” in Phytomicrobiome and Stress Regulation, Amsterdam, Netherlands: Elsevier, 351–370.

[ref37] Jack A. L. H. Nelson E. B. (2018). A seed-recruited microbiome protects developing seedlings from disease by altering homing responses of *Pythium aphanidermatum* zoospores. Plant Soil 422, 209–222. doi: 10.1007/S11104-017-3257-2

[ref38] Jacoby R. Peukert M. Succurro A. Koprivova A. Kopriva S. (2017). The role of soil microorganisms in plant mineral nutrition—current knowledge and future directions. Front. Plant Sci. 8:292271. doi: 10.3389/FPLS.2017.01617/XML PMC5610682 28974956

[ref39] Jha R. Manonmani V. Sundaralingam K. Vanitha S. Gnanachitra M. Kalaiselvi T. . (2025). The seed microbiome: microbial hashes for plant wellbeing. Open J. Environ. Biol. 10, 007–022. doi: 10.17352/OJEB.000046

[ref40] Johnston-Monje D. Gutiérrez J. P. Lopez-Lavalle L. A. B. (2021). Seed-transmitted bacteria and fungi dominate juvenile plant microbiomes. Front. Microbiol. 12:737616. doi: 10.3389/FMICB.2021.737616/TEXT 34745040 PMC8569520

[ref41] Kabato W. S. Hailegnaw N. Chaffamo T. E. Samuel A. De Silva A. G. S. D. Molnár Z. (2025). Microalgae-based strategies for soil health and crop productivity: mechanisms, challenges, and pathways to climate-resilient agriculture. Agronomy 15:2669. doi: 10.3390/AGRONOMY15112669

[ref42] Khawula S. Daniel A. I. Nyawo N. Ndlazi K. Sibiya S. Ntshalintshali S. . (2025). Optimizing plant resilience with growth-promoting Rhizobacteria under abiotic and biotic stress conditions. Plant Stress 17:100949. doi: 10.1016/J.STRESS.2025.100949

[ref43] Kim H. Jeon J. Lee K. K. Lee Y. H. (2022). Longitudinal transmission of bacterial and fungal communities from seed to seed in rice. Commun. Biol. 5:5. doi: 10.1038/s42003-022-03726-w, 35915150 PMC9343636

[ref44] Kong H. G. Song G. C. Ryu C. M. (2019). Inheritance of seed and rhizosphere microbial communities through plant–soil feedback and soil memory. Environ. Microbiol. Rep. 11, 479–486. doi: 10.1111/1758-2229.12760 31054200

[ref45] Krishnan R. Menon R. R. Tanaka N. Busse H. J. Krishnamurthi S. Rameshkumar N. (2016). Arthrobacter pokkalii sp nov, a novel plant associated actinobacterium with plant beneficial properties, isolated from saline tolerant Pokkali rice, Kerala, India. PLoS One 11:e0150322. doi: 10.1371/JOURNAL.PONE.0150322, 26963092 PMC4786123

[ref46] Lal R. Monger C. Nave L. Smith P. (2021). The role of soil in regulation of climate. Philos. Trans. R. Soc. Lond. Ser. B Biol. Sci. 376:20210084. doi: 10.1098/RSTB.2021.0084, 34365818 PMC8349633

[ref47] Li H. P. Ma H. B. Zhang J. L. (2025). Halo-tolerant plant growth-promoting bacteria-mediated plant salt resistance and microbiome-based solutions for sustainable agriculture in saline soils. FEMS Microbiol. Ecol. 101:fiaf037. doi: 10.1093/FEMSEC/FIAF037, 40194942 PMC12051855

[ref48] Liu Q. Zhao W. (2023). Plant–soil microbe feedbacks drive seedling establishment during secondary forest succession: the ‘successional stage hypothesis.’. J. Plant Ecol. 16:rtad021. doi: 10.1093/JPE/RTAD021

[ref49] Liu J. Zhao X. Niu Y. Ren Y. Wang M. Han B. . (2025). Plant growth-promoting rhizobacteria Halomonas alkaliantarcticae M23 promotes the salt tolerance of maize by increasing the K+/Na+ ratio, antioxidant levels, and ABA levels and changing the rhizosphere bacterial community. BMC Plant Biol. 25:727. doi: 10.1186/S12870-025-06765-7/FIGURES/7 40442582 PMC12121225

[ref50] Loiko N. Islam M. N. (2024). Plant–soil microbial interaction: differential adaptations of beneficial vs. pathogenic bacterial and fungal communities to climate-induced drought. Agronomy 14:1949. doi: 10.3390/AGRONOMY14091949

[ref51] Luo H. Win C. S. Lee D. H. He L. Yu J. M. (2024). *Microbacterium azadirachtae* CNUC13 enhances salt tolerance in maize by modulating osmotic and oxidative stress. Biology 13:244. doi: 10.3390/BIOLOGY13040244, 38666856 PMC11048422

[ref52] Lyu X. Nuhu M. Candry P. Wolfanger J. Betenbaugh M. Saldivar A. . (2024). Top-down and bottom-up microbiome engineering approaches to enable biomanufacturing from waste biomass. J. Ind. Microbiol. Biotechnol. 51:kuae025. doi: 10.1093/JIMB/KUAE025, 39003244 PMC11287213

[ref53] Martins M. R. Jantalia C. P. Reis V. M. Döwich I. Polidoro J. C. Alves B. J. R. . (2018) Impact of plant growth-promoting bacteria on grain yield, protein content, and urea-^15^ n recovery by maize in a Cerrado Oxisol Plant Soil 422;239–250. doi: 10.1007/S11104-017-3193-1

[ref54] Marzouk S. H. Kwaslema D. R. Omar M. M. Mohamed S. H. (2025). “Harnessing the power of soil microbes: their dual impact in integrated nutrient management and mediating climate stress for sustainable rice crop production” a systematic review. Heliyon 11:e41158. doi: 10.1016/J.HELIYON.2024.E41158, 39758363 PMC11699367

[ref55] Matsumoto H. Fan X. Wang Y. Kusstatscher P. Duan J. Wu S. . (2021). Bacterial seed endophyte shapes disease resistance in rice. Nat. Plants 7, 60–72. doi: 10.1038/S41477-020-00826-5, 33398157

[ref56] Mishra P. Mishra J. Arora N. K. (2021). Plant growth promoting bacteria for combating salinity stress in plants - recent developments and prospects: a review. Microbiol. Res. 252:126861. doi: 10.1016/J.MICRES.2021.126861, 34521049

[ref57] Mrunalini K. Behera B. Jayaraman S. Abhilash P. C. Dubey P. K. Swamy G. N. . (2022). Nature-based solutions in soil restoration for improving agricultural productivity. LDeDe 33, 1269–1289. doi: 10.1002/LDR.4207

[ref58] Nam N. N. Do H. D. K. Loan Trinh K. T. Lee N. Y. (2023). Metagenomics: An effective approach for exploring microbial diversity and functions. Foods 12:2140. doi: 10.3390/FOODS12112140 37297385 PMC10252221

[ref59] Neale D. Cullen L. Ranout A. S. (2024). Improving soil health in the UK: why a microbial approach is indispensable in attaining sustainable soils. Sustain. Microbiol. 1: qvae026. doi: 10.1093/SUMBIO/QVAE026 PMC13375347 42576854

[ref60] Neuenkamp L. García de León D. Hamer U. Hölzel N. McGale E. Hannula S. E. (2024). Comprehensive tools for ecological restoration of soils foster sustainable use and resilience of agricultural land. Commun. Biol. 7:1577. doi: 10.1038/S42003-024-07275-2, 39592854 PMC11599581

[ref61] Noman M. Ahmed T. Ijaz U. Shahid M. Azizullah L. Manzoor I. . (2021). Plant–microbiome crosstalk: dawning from composition and assembly of microbial community to improvement of disease resilience in plants. Int. J. Mol. Sci. 22:6852. doi: 10.3390/IJMS22136852 34202205 PMC8269294

[ref62] Núñez-Cano J. Ruiz-Castilla F. J. Ramos J. Romera F. J. Lucena C. (2025). *Debaryomyces hansenii* enhances growth, nutrient uptake, and yield in Rice plants (*Oryza sativa* L.) cultivated in calcareous soil. Agronomy 15:1696. doi: 10.3390/AGRONOMY15071696 PMC12737272 41470650

[ref63] O’Callaghan M. (2016). Microbial inoculation of seed for improved crop performance: issues and opportunities. Appl. Microbiol. Biotechnol. 100, 5729–5746. doi: 10.1007/S00253-016-7590-9, 27188775 PMC4909795

[ref64] Pandey K. Saharan B. S. (2025). Soil microbiomes: a promising strategy for boosting crop yield and advancing sustainable agriculture. Discover Agriculture 3:54. doi: 10.1007/S44279-025-00208-5

[ref65] Patel P. Gajjar H. Joshi B. Krishnamurthy R. Amaresan N. (2022). Inoculation of salt-tolerant Acinetobacter sp (RSC9) improves the sugarcane (Saccharum sp. hybrids) growth under salinity stress condition. Sugar Tech 24, 494–501. doi: 10.1007/S12355-021-01043-W

[ref66] Peng J. Zhou X. Rensing C. Liesack W. Zhu Y. G. (2024). Soil microbial ecology through the lens of metatranscriptomics. Soil Ecol. Letters 6:230217. doi: 10.1007/S42832-023-0217-Z

[ref67] Portal-Gonzalez N. Wang W. He W. Santos-Bermudez R. (2025). Engineering plant holobionts for climate-resilient agriculture. ISME J. 19:wraf158. doi: 10.1093/ISMEJO/WRAF158, 40748243 PMC12381762

[ref68] Priyadarshini C. Lal R. Yuan P. Liu W. Adhikari A. Bhandari S. . (2025). Plant disease Suppressiveness enhancement via soil health management. Biology 14:924. doi: 10.3390/BIOLOGY14080924 40906075 PMC12383925

[ref69] Renganathan P. Gaysina L. A. Rueda-Puente E. O. (2025). Microbial biofertilizers for salinity stress mitigation in hydroponic systems. Curr. Issues Mol. Biol. 47:1029. doi: 10.3390/CIMB47121029, 41614794 PMC12731808

[ref70] Rétif F. Kunz C. Calabro K. Duval C. Prado S. Bailly C. . (2023). Seed fungal endophytes as biostimulants and biocontrol agents to improve seed performance. Front. Plant Sci. 14:1260292. doi: 10.3389/FPLS.2023.1260292/XML, 37941673 PMC10628453

[ref71] Rochefort A. Simonin M. Marais C. Guillerm-Erckelboudt A.-Y. Barret M. Sarniguet A. (2021). Transmission of seed and soil microbiota to seedling. mSystems 6:e00446-21. doi: 10.1128/MSYSTEMS.00446-21, 34100639 PMC8269233

[ref72] Romão I. R. Do J. Gomes C. Silva D. Ignacio Vilchez J. (2025). The seed microbiota from an application perspective: an underexplored frontier in plant–microbe interactions. Crop Health 3:12. doi: 10.1007/S44297-025-00051-6 41649661 PMC12825913

[ref73] Santoyo G. Guzmán-Guzmán P. Parra-Cota F. I. de los Santos-Villalobos S. Orozco-Mosqueda M. D. C. Glick B. R. (2021). Plant growth stimulation by microbial consortia. Agronomy 11:219. doi: 10.3390/AGRONOMY11020219

[ref74] Sarhan M. S. Hamza M. A. Youssef H. H. Patz S. Becker M. ElSawey H. . (2019). Culturomics of the plant prokaryotic microbiome and the dawn of plant-based culture media – a review. J. Adv. Res. 19, 15–27. doi: 10.1016/J.JARE.2019.04.002, 31341666 PMC6630032

[ref75] Schmidt J. E. Kent A. D. Brisson V. L. Gaudin A. C. M. (2019). Agricultural management and plant selection interactively affect rhizosphere microbial community structure and nitrogen cycling. Microbiome 7:146. doi: 10.1186/S40168-019-0756-9/FIGURES/6 31699148 PMC6839119

[ref76] Shade A. Jacques M. A. Barret M. (2017). Ecological patterns of seed microbiome diversity, transmission, and assembly. Curr. Opin. Microbiol. 37, 15–22. doi: 10.1016/J.MIB.2017.03.010, 28437661

[ref77] Shahid M. Al-Khattaf F. S. Danish M. Zeyed M. T. Atef Hatamleh A. Mohamed A. . (2022). PGPR Kosakonia Radicincitans KR-17 increases the salt tolerance of radish by regulating ion-homeostasis, photosynthetic molecules, redox potential, and stressor metabolites. Front. Plant Sci. 13:919696. doi: 10.3389/FPLS.2022.919696/XML 35979076 PMC9376370

[ref78] Shahid M. Singh U. B. Khan M. S. Singh P. Kumar R. Singh R. N. . (2023). Bacterial ACC deaminase: insights into enzymology, biochemistry, genetics, and potential role in amelioration of environmental stress in crop plants. Front. Microbiol. 14:1132770. doi: 10.3389/FMICB.2023.1132770/XML 37180266 PMC10174264

[ref79] Sharma A. Bora P. (2025). Engineering synthetic microbial communities to restructure the phytobiome for plant health and productivity. World J. Microbiol. Biotechnol. 41:228. doi: 10.1007/S11274-025-04460-1, 40560276

[ref80] Sharma A. Das N. Pandey P. Shukla P. (2025). Plant-microbiome responses under drought stress and their metabolite-mediated interactions towards enhanced crop resilience. Curr. Plant Biol. 43:100513. doi: 10.1016/J.CPB.2025.100513

[ref81] Shrivastava P. Kumar R. (2015). Soil salinity: a serious environmental issue and plant growth promoting bacteria as one of the tools for its alleviation. Saudi J. Biol. Sci. 22, 123–131. doi: 10.1016/J.SJBS.2014.12.001, 25737642 PMC4336437

[ref82] Singh A. Mazahar S. Chapadgaonkar S. S. Giri P. Shourie A. (2023). Phyto-microbiome to mitigate abiotic stress in crop plants. Front. Microbiol. 14:1210890. doi: 10.3389/FMICB.2023.1210890, 37601386 PMC10433232

[ref83] Sliti A. Singh V. Pande A. Shin J. H. (2025). Soil holobiont interplay and its role in protecting plants against salinity stress. Pedosphere 35, 97–115. doi: 10.1016/J.PEDSPH.2024.09.002

[ref84] Srivastava S. Srivastava S. (2020). Prescience of endogenous regulation in *Arabidopsis thaliana* by *Pseudomonas putida* MTCC 5279 under phosphate starved salinity stress condition. Sci. Rep. 10:5855. doi: 10.1038/s41598-020-62725-1, 32246044 PMC7125087

[ref85] Srivastava S. Tyagi R. Sharma S. (2024). Seed biopriming as a promising approach for stress tolerance and enhancement of crop productivity: a review. J. Sci. Food Agric. 104, 1244–1257. doi: 10.1002/JSFA.13048, 37824780

[ref86] Streletskii R. A. Astaykina A. A. Belov A. A. Cheptsov V. S. Vetrova A. A. (2024). “Beneficial soil microorganisms and their role in sustainable agriculture,” in Sustainable Agricultural Practices, Leeds, United Kingdom: Emerald Publishing Limited. 293–333.

[ref87] Sulesky-Grieb A. Simonin M. Bintarti A. F. Marolleau B. Barret M. Shade A. (2024). Stable, multigenerational transmission of the bean seed microbiome despite abiotic stress. mSystems 9:e0095124. doi: 10.1128/msystems.00951-24 39475253 PMC11575401

[ref88] Tang L. Zhan L. Han Y. Wang Z. Dong L. Zhang Z. (2023). Microbial community assembly and functional profiles along the soil-root continuum of salt-tolerant Suaeda glauca and Suaeda salsa. Front. Plant Sci. 14:1301117. doi: 10.3389/FPLS.2023.1301117/BIBTEX, 38046600 PMC10691491

[ref89] Țopa D. C. Căpșună S. Calistru A. E. Ailincăi C. (2025). Sustainable practices for enhancing soil health and crop quality in modern agriculture: a review. Agriculture 15, 998–998. doi: 10.3390/AGRICULTURE15090998

[ref90] Topçuoğlu B. D. Lesniak N. A. Ruffin M. T. Wiens J. Schloss P. D. (2020). A framework for effective application of machine learning to microbiome-based classification problems. MBio 11:e00434–20. doi: 10.1128/MBIO.00434-20, 32518182 PMC7373189

[ref91] Trivedi P. Leach J. E. Tringe S. G. Sa T. Singh B. K. (2020). Plant–microbiome interactions: from community assembly to plant health. Nat. Rev. Microbiol. 18, 607–621. doi: 10.1038/s41579-020-0412-1, 32788714

[ref92] Vannier N. Mesny F. Getzke F. Chesneau G. Dethier L. Ordon J. . (2023). Genome-resolved metatranscriptomics reveals conserved root colonization determinants in a synthetic microbiota. Nat. Commun. 14:8274. doi: 10.1038/S41467-023-43688-Z, 38092730 PMC10719396

[ref93] Vannier N. Mony C. Bittebiere A.-K. Theis K. R. Rosenberg E. Vandenkoornhuyse P. (2019). Clonal plants as meta-holobionts. mSystems 4:e00213-18. doi: 10.1128/MSYSTEMS.00213-18, 30944875 PMC6426648

[ref94] Vincze É. B. Becze A. Laslo É. Mara G. (2024). Beneficial soil microbiomes and their potential role in plant growth and soil fertility. Agriculture 14, 152–152. doi: 10.3390/AGRICULTURE14010152

[ref95] Wang X. Chi Y. Song S. (2024). Important soil microbiota’s effects on plants and soils: a comprehensive 30-year systematic literature review. Front. Microbiol. 15:1347745. doi: 10.3389/FMICB.2024.1347745, 38591030 PMC10999704

[ref96] Zeng Q. Zhao Y. Shen W. Han D. Yang M. (2023). Seed-to-seed: plant Core vertically transmitted microbiota. J. Agric. Food Chem. 71, 19255–19264. doi: 10.1021/ACS.JAFC.3C07092, 38044571

[ref97] Zhou H. Shi H. Yang Y. Feng X. Chen X. Xiao F. . (2024). Insights into plant salt stress signaling and tolerance. J. Genet. Genomics 51, 16–34. doi: 10.1016/J.JGG.2023.08.007, 37647984

